# Supplementary material for: A point mutation in recC associated with subclonal replacement of carbapenem-resistant Klebsiella pneumoniae ST11 in China
Source: Nat Commun. 2023 Apr 28;14:2464. doi: 10.1038/s41467-023-38061-z (PMC10147710; doi:10.1038/s41467-023-38061-z)
Supplement: Supplementary file 1 — Supplementary figures [file 41467_2023_38061_MOESM1_ESM.pdf]

Supplementary Materials for

Subclonal replacement of carbapenem-resistant *Klebsiella pnuemoniae* ST11 causing bloodstream infection is driven by a selective population with enhanced inter-hospital transmission in China

Kai Zhou1[*†], Chun-xu Xue[1†], Tingting Xu[1†], Ping Shen[2†], Sha Wei[1], Kelly L. Wyres[3], Margaret M. C. Lam[3], Jinquan Liu[1], Haoyun Lin[4], Yunbo Chen[2], Kathryn E. Holt[3,5], the BRICS Working Group and Yonghong Xiao[2*]

*Corresponding author. Email: zhouk@mail.sustech.edu.cn; xiaoyonghong@zju.edu.cn

the BRICS Working Group

Yunbo Chen (The First Affiliated Hospital of Zhejiang University), Hui Ding (Lishui City Central Hospital), Yongyun Liu (Affiliated Hospital of Binzhou Medical College), Haifeng Mao (the First People's Hospital of Lianyungang), Ying Huang (First Affiliated Hospital of Anhui Medical University), Zhenghai Yang (Yijishan Hospital of Wannan Medical College), Yuanyuan Dai (Anhui Provincial Hospital), Guolin Liao (Wuhan Puren Hospital), Lisha Zhu (The First People's Hospital of Jingzhou), Liping Zhang (People's Hospital of Ningxia Hui Autonomous Region), Yanhong Li (Anyang District Hospital of Henan Province), Hongyun Xu (The Second People's Hospital of Yunnan Province), Junmin Cao (Zhejiang Provincial Hospital of Traditional Chinese Medicine), Baohua Zhang (People's Hospital of Huangshan City), Liang Guo (Mindong Hospital of Ningde City), Haixin Dong (The Affiliated Hospital of Jining Medical University), Shuyan Hu (People's Hospital of Qingyang), Sijin Man (Tengzhou Centre People's Hospital), Lu Wang (Lu'an People's Hospital), Zhixiang Liao (Xinjiang Uygur Autonomous Region Youyi Hospital), Rong Xu (People's Hospital of Yichun City), Dan Liu (Jiujiang First People's Hospital), Yan Jin (Shandong Provincial Hospital), Yizheng Zhou (Jingzhou Central Hospital), Yiqun Liao (the First Affiliated Hospital of Gannan Medical University), Fenghong Chen (The First Hospital of Putian City), Beiqing Gu (People's Hospital of Haining City), Jiliang Wang (Shengli Oilfield Central Hospital), Jinhua Liang (The Affiliated Hongqi Hospital of Mudanjiang Medicine College), Lin Zheng (The Affiliated Hospital of Ningbo Medical School), Aiyun Li (Women's Hospital, Zhejiang University School of Medicine), Jilu Shen (The Fourth Affiliated Hospital of Anhui Medical University), Yinqiao Dong (Tianchang City People's Hospital), Lixia Zhang (Shanxi Provincial People's Hospital), Hongxia Hu (The First Affiliated Hospital of Henan University of Science and Technology), Bo Quan (The Second People's Hospital of Jingzhou), Wencheng Zhu (Lu'an Civily Hospital), Kunpeng Liang (the Second Affiliated Hospital of Bengbu Medicine College), Qiang Liu (Huaihe Hospital of Henan University), Shifu Wang (Qilu Children's Hospital of Shandong University), Xiaoping Yan (Zigong Third People's

Chuandan Wan (Changshu Medicine Examination Institute), Xiaoyan Qi (Women and Children's Hospital of Jin'an District), Fei Du (Hubin Hospital of Hefei)

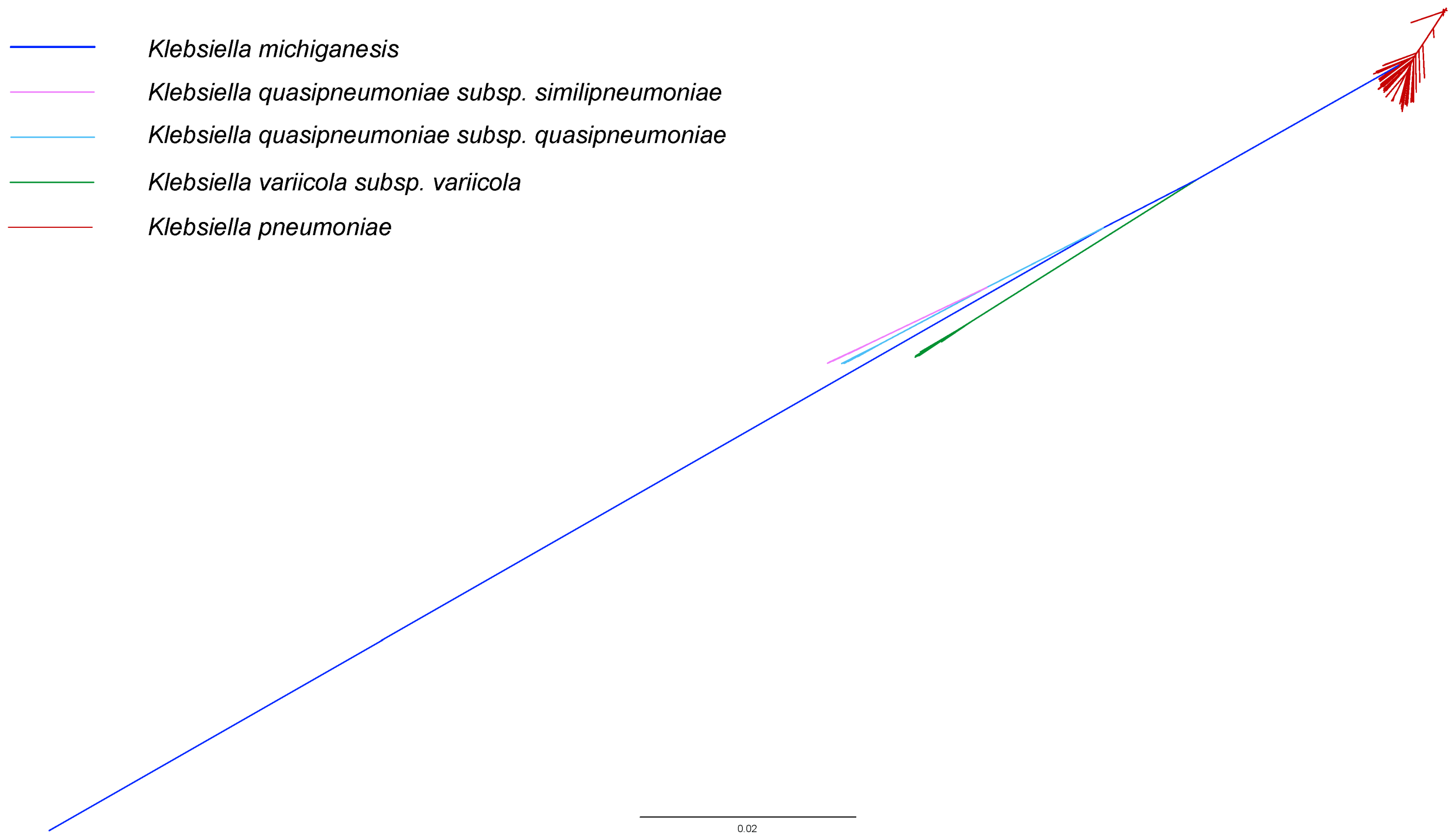

Fig. S1. A phylogenetic tree of the 794 CRKP isolates analysed in this study.

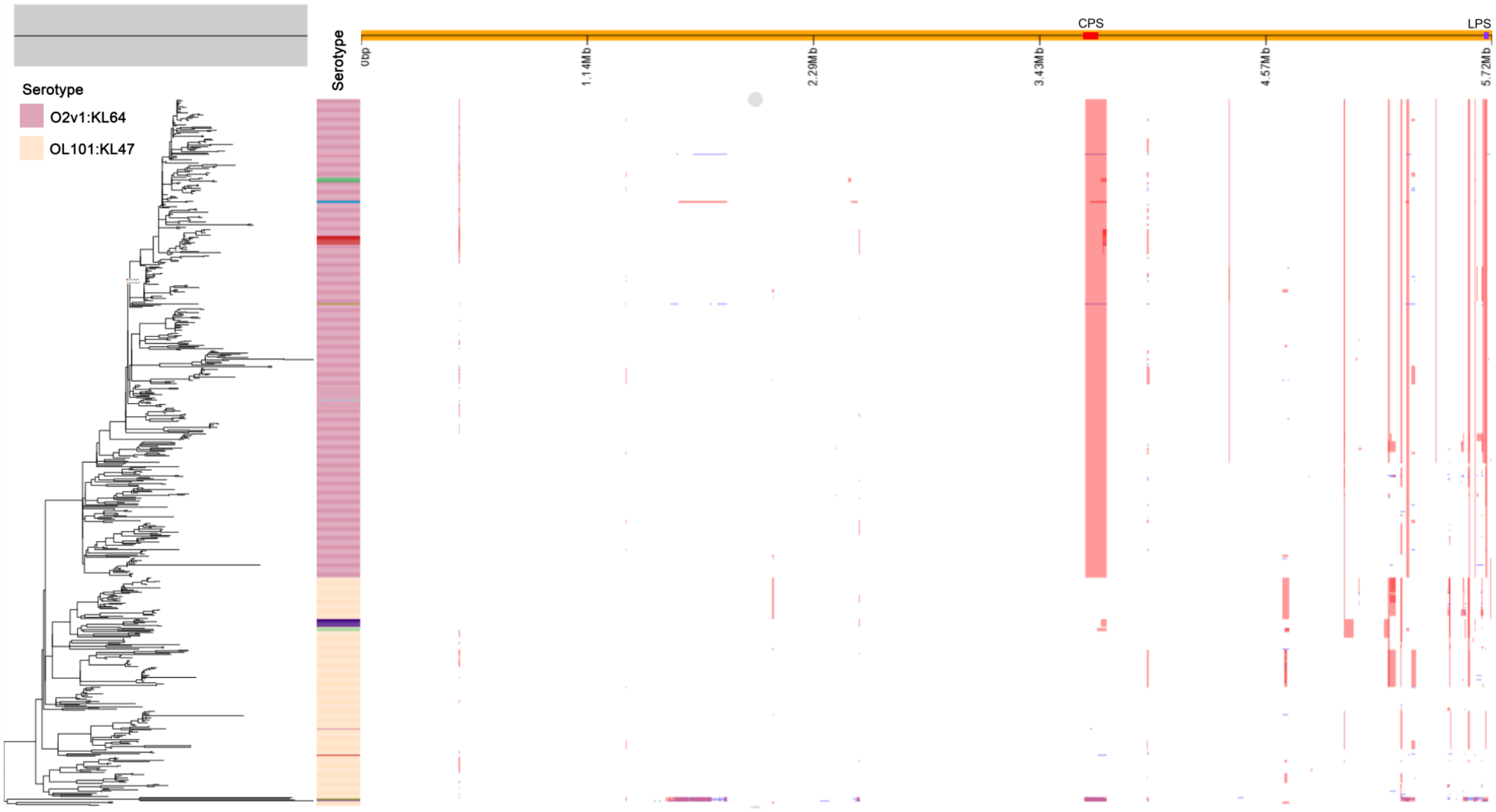

Fig. S2. Recombinant genomic regions detected among the 646 CRKP-ST11 isolates. Recombinant genomic regions were predicted by Gubbinsusing an OL101:KL47 genome (KP29407) as the reference. The loci of CPS and LPS are indicated on the reference. Figure was produced by Phandango.

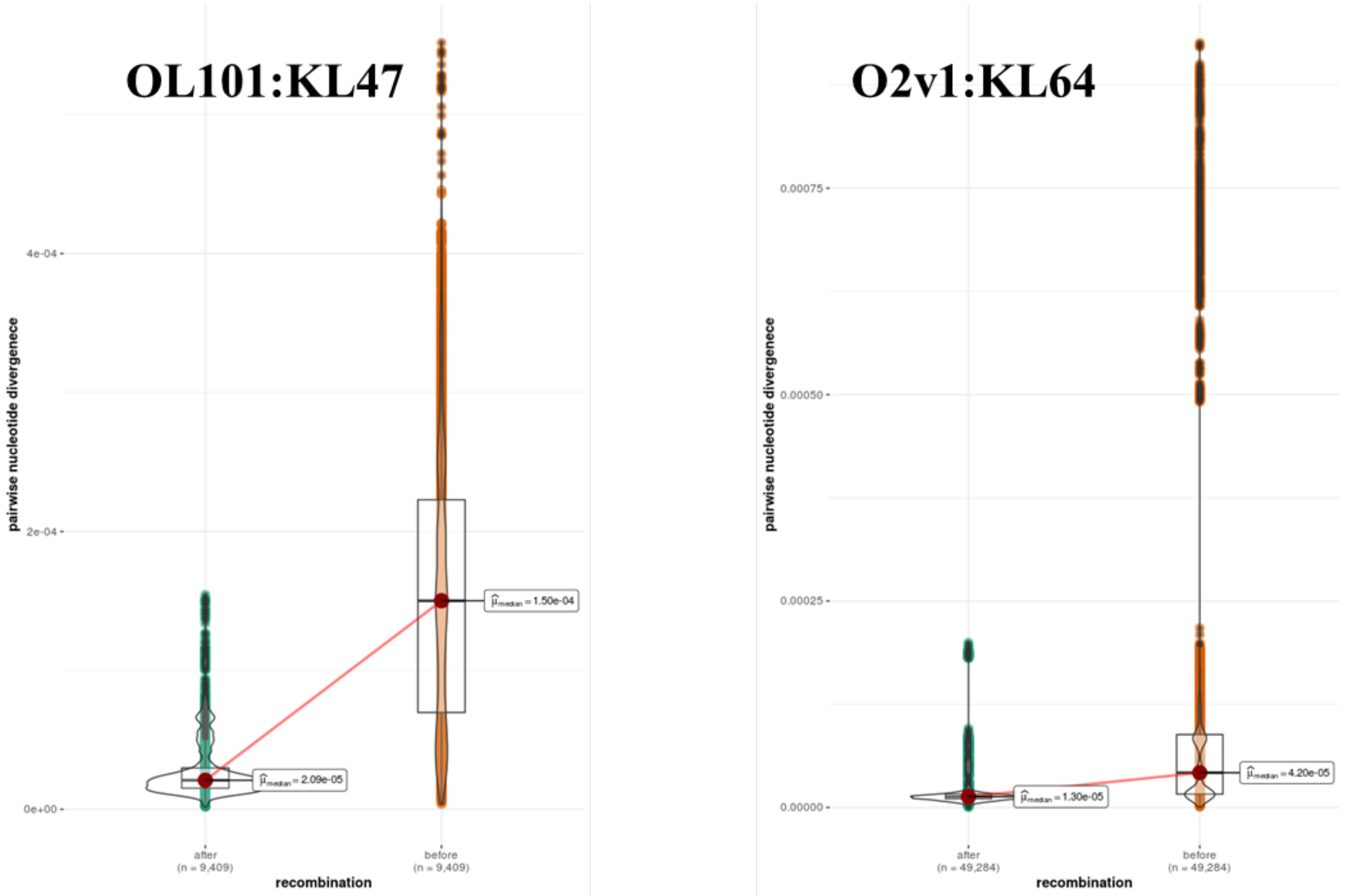

Fig. S3. Distribution of pairwise nucleotide divergence for all genomes within O2v1:KL64 and OL101:KL47 before and after the removal of recombinant sequence regions. Distribution of pairwise nucleotide divergence detected in O2v1:KL64 (n = 422) and OL101:KL47 (n = 192) before and after the removal of recombinant sequence regions were significantly different ($p < 2.2x10^{-16}$), respectively. Boxplots are displayed using the Tukey method (centre line, median; box limits, upper and lower quartiles; whiskers, last point within a 1.5x interquartile range).

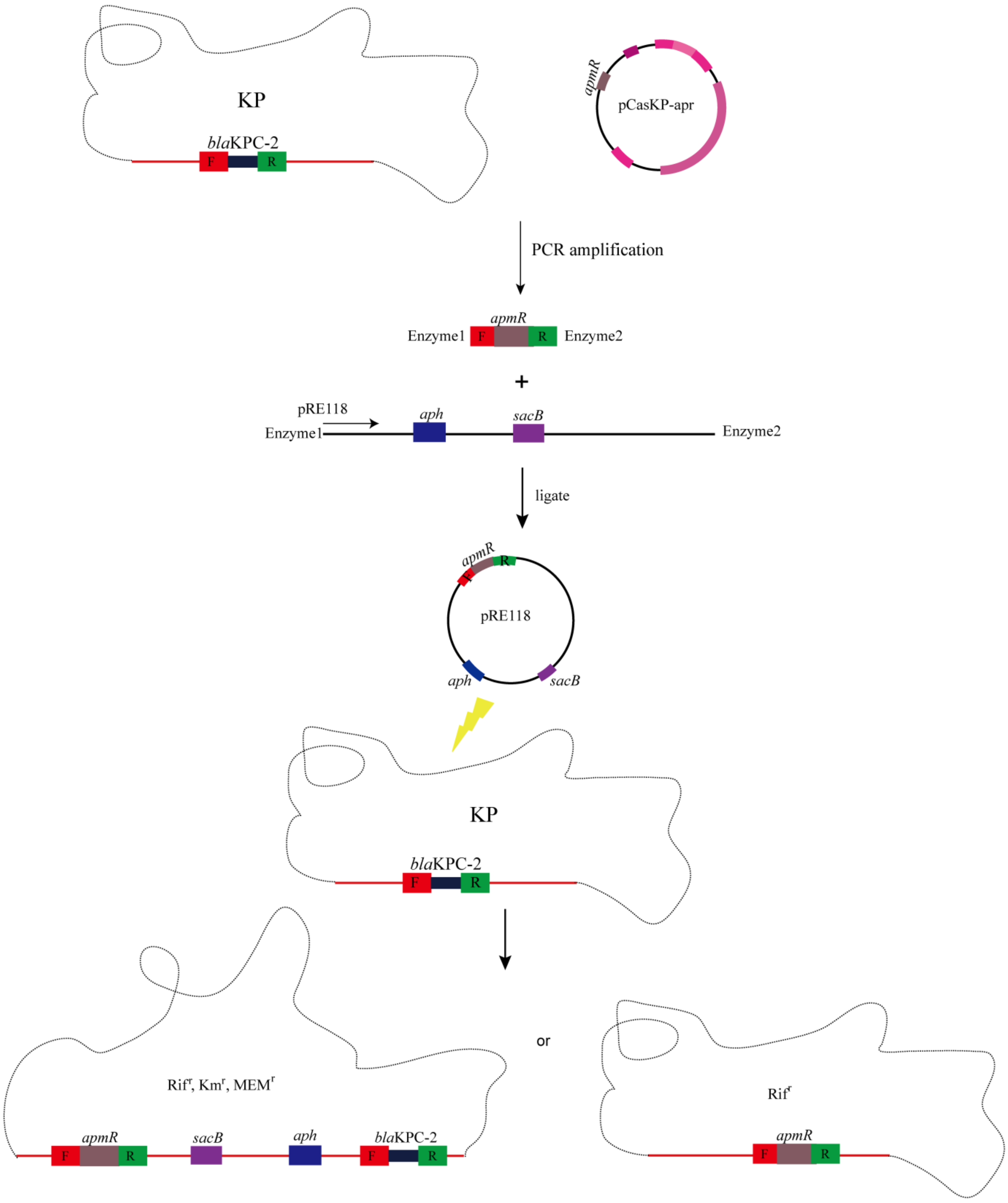

Fig. S4. Scheme of the transduction experiment to measure recombination frequency. The upstream and downstream fragment of *bla*$_{\mathrm{KPC\text{-}2}}$ and an apramycin resistance gene *apmR* amplified from KP37485 and pCasKP-apr, respectively, was ligated and cloned into pRE118. The resultant plasmid was electroporated into KP37485. Recombinants were selected on 50 mg/L apramycin-containing plates. Two types of recombinants were generated by the assay.

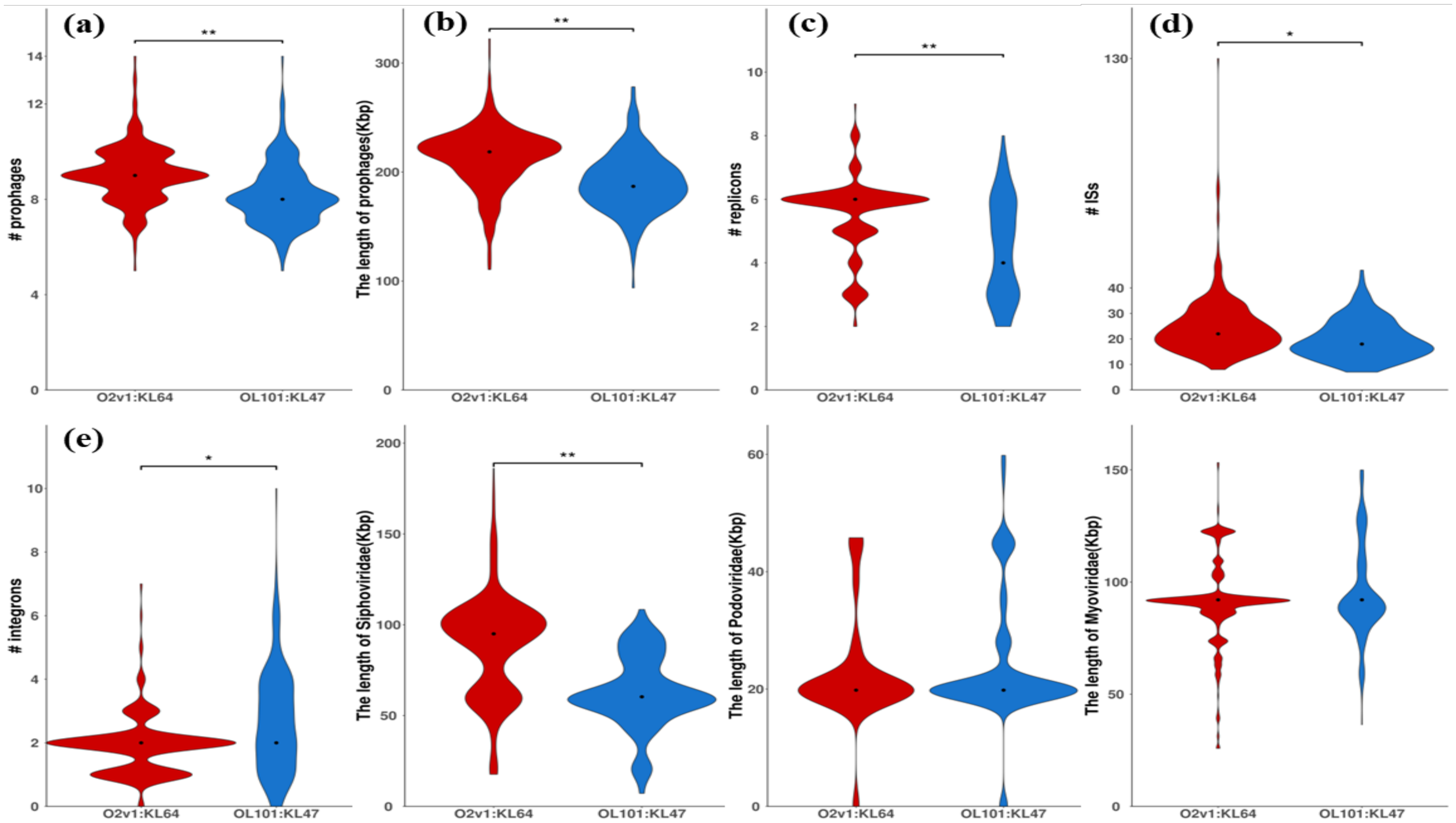

Fig. S5. Diversity of MGEs between O2v1:KL64 and OL101:KL47. a-b, Violin plots showing the distributions of the number of prophages ($p < 2.2\times10^{-16}$ by Wilcoxon Rank Sum test) (a) and the total length of prophage sequence ($p < 2.2\times10^{-16}$ by Wilcoxon Rank Sum test) (b) identified per genome. c-e, Violin plots showing the distributions of plasmid replicon ($p < 2.2\times10^{-16}$ by Wilcoxon Rank Sum test) (c), IS copy numbers ($p = 1.4\times10^{-13}$ by Wilcoxon Rank Sum test) (d) and integrons ($p = 6.9\times10^{-9}$ by Wilcoxon Rank Sum test) (e) count per genome. f-h, The prophages detected are classified into three families, namely *Myoviridae*, *Podoviridae*, and *Siphoviridae*. Violin plots showing the total length of *Siphoviridae* ($p < 2.2\times10^{-16}$ by Wilcoxon Rank Sum test) (f), *Podoviridae* ($p = 0.00022$ by Wilcoxon Rank Sum test) (g) and *Myoviridae* ($p = 0.0047$ by Wilcoxon Rank Sum test) (h) sequence identified per genome. For all panels, brackets indicate two-sided Wilcoxon Rank Sum tests of pairwise group comparisons; ns, not significant; *, $p < 0.05$; **, $p < 1\times10^{-15}$.

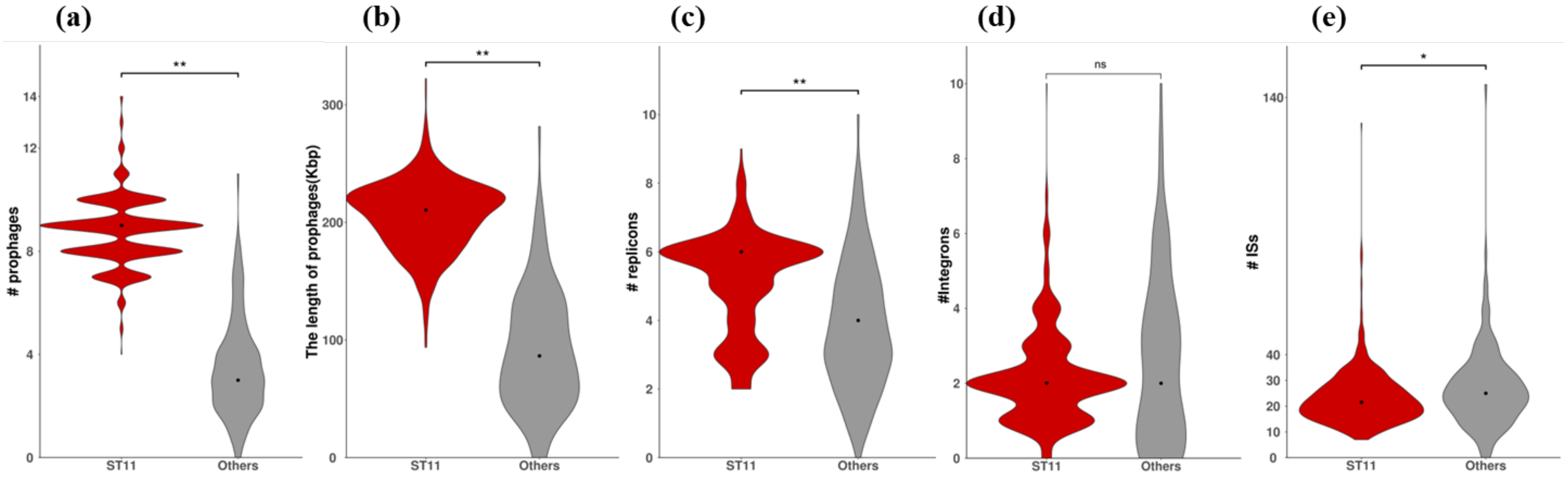

Fig. S6. Diversity of MGEs between ST11 and non-ST11 isolates collected in this study. a-b**,** Violin plots showing the distributions of the number of prophages ($p < 2.2 \times 10^{-16}$ by Wilcoxon Rank Sum test) (a) and the total length of prophage sequence ($p < 2.2 \times 10^{-16}$ by Wilcoxon Rank Sum test) (b) identified per genome. c-e, Violin plots showing the distributions of plasmid replicon ($p < 2.2 \times 10^{-16}$ by Wilcoxon Rank Sum test) (c), integrons ($p = 0.33$ by Wilcoxon Rank Sum test) (d) and IS copy numbers ($p = 0.0026$ by Wilcoxon Rank Sum test) (e) count per genome. For all panels, brackets indicate two-sided Wilcoxon Rank Sum tests of pairwise group comparisons; ns, not significant; *, $p < 0.05$; **, $p < 1 \times 10^{-15}$.

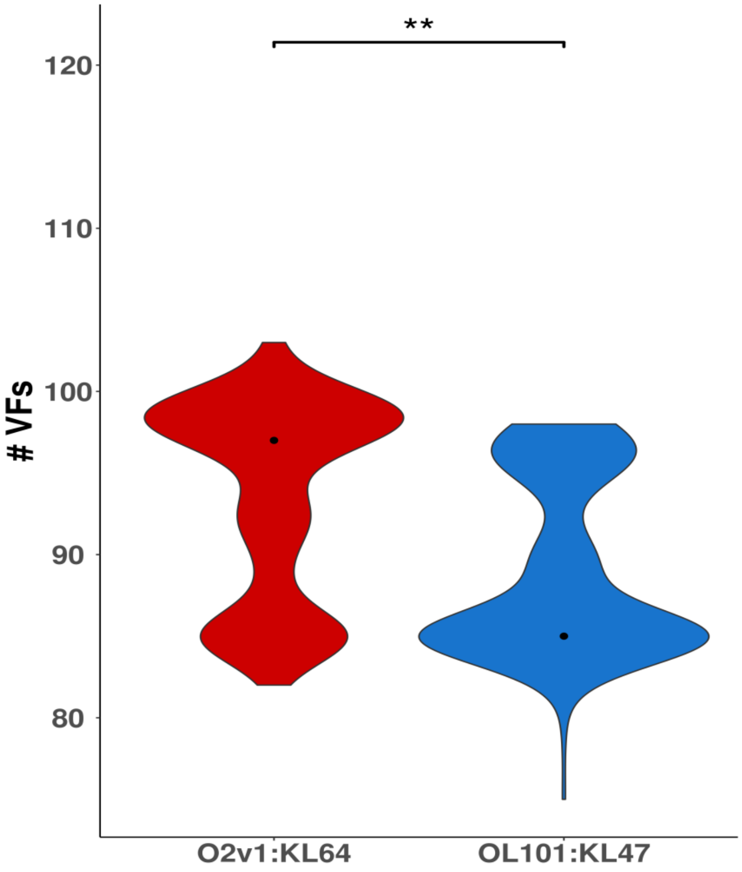

Fig. S7. Distribution of 154 VFs in O2v1:KL64 and OL101:KL47. The bracket indicates Wilcoxon Rank Sum test of pairwise group comparison; **, $p < 2.2\times10^{-16}$ (by two-sided Wilcoxon Rank Sum test).

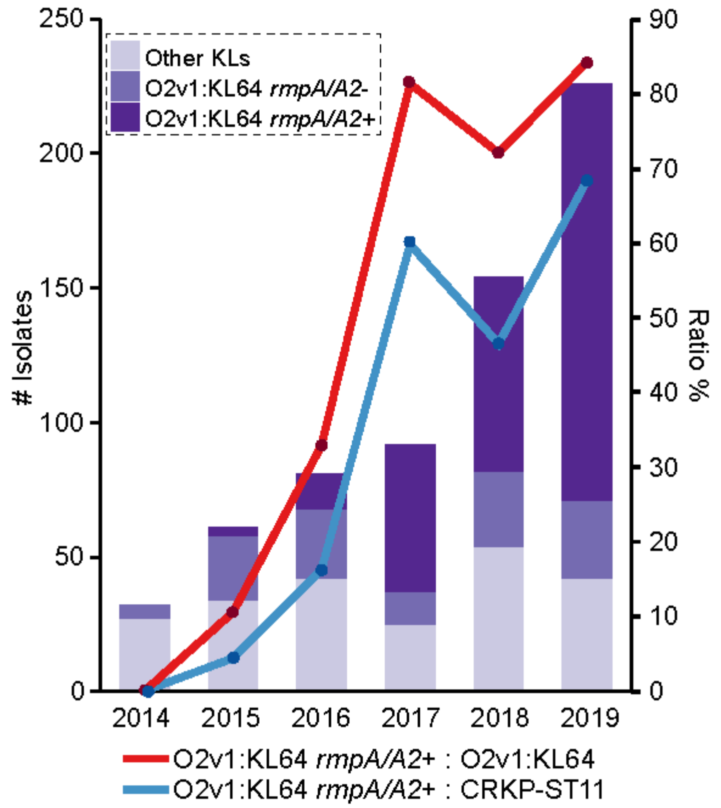

Fig. S8. Distribution of O2v1:KL64-hvKP in CRKP-ST11 during the surveillance. The graph shows the number of O2v1:KL64-hvKP, O2v1:KL64-others and the other subclones detected in CRKP-ST11 each year. The lines indicate the ratio of O2v1:KL64-hvKP/O2v1:KL64 (red) and O2v1:KL64-hvKP/CRKP-ST11 each year (blue)

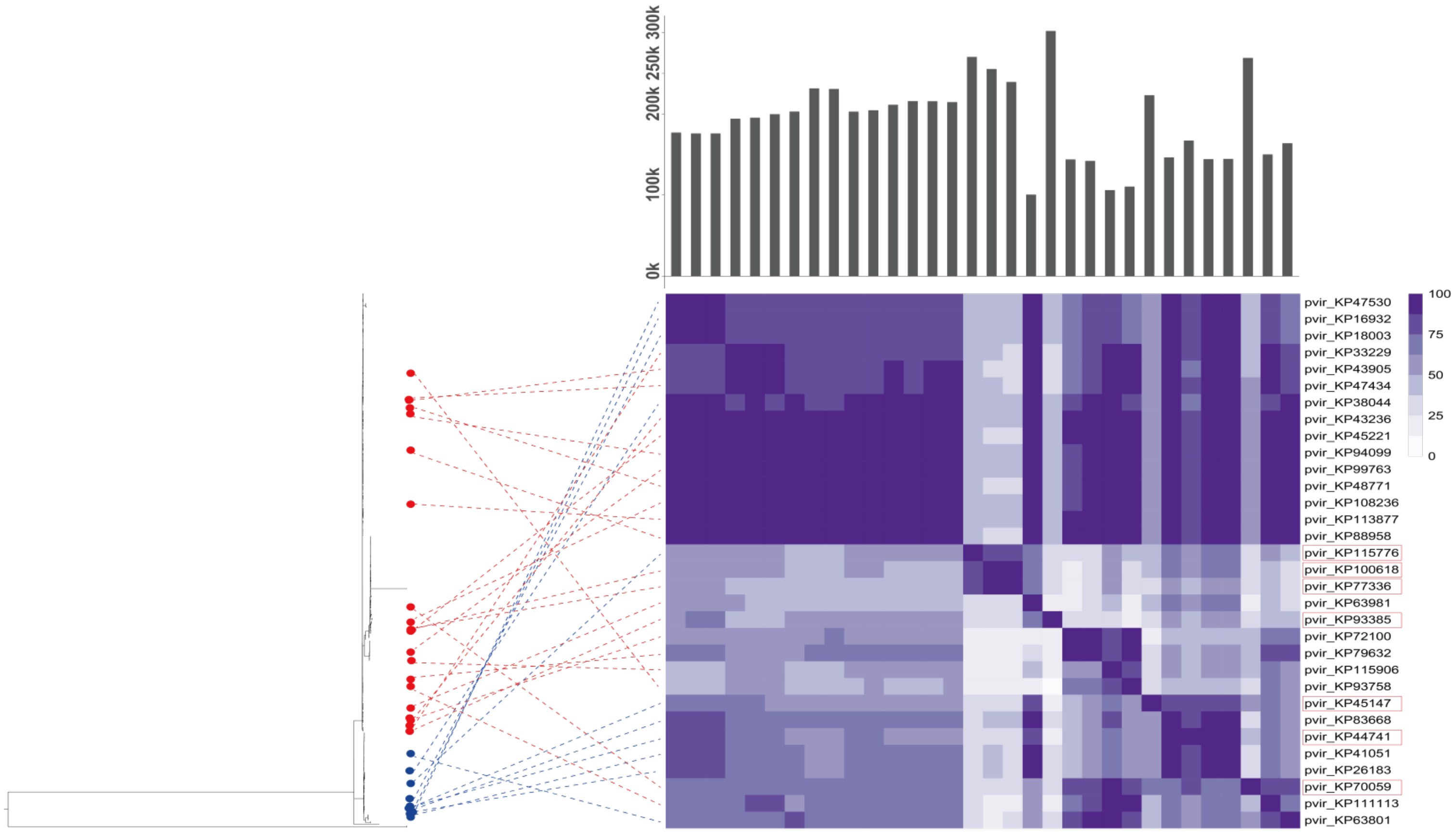

Fig. S9. Pairwise comparison of 32 (putative) virulence plasmids. The comparison includes 30 (putative) virulence plasmids sequenced in this study and 2 reference virulence plasmids (pVir-KP16932 and pVir-KP47434). Twenty-five of the 30 virulence plasmids share a relatively conserved backbone, and the others framed are fusion plasmids. The phylogenetic tree contains all *rmpA/A2*-positive ST11 isolates with the 2 references (KP16932 and KP47434). Isolates of O2v1:KL64 (n=20) and OL101:KL47 (n=10) from which (putative) virulence plasmids were obtained are marked by red and blue circles in the tree, respectively. The heat map shows the percentage of bases in each plasmid that could be aligned to each of the other plasmids (the row and column orders are the same). Dotted lines link the long read-sequenced isolates in the phylogenetic tree to their respective plasmids in the heat map. The length of each plasmid is shown by a bar on the top.

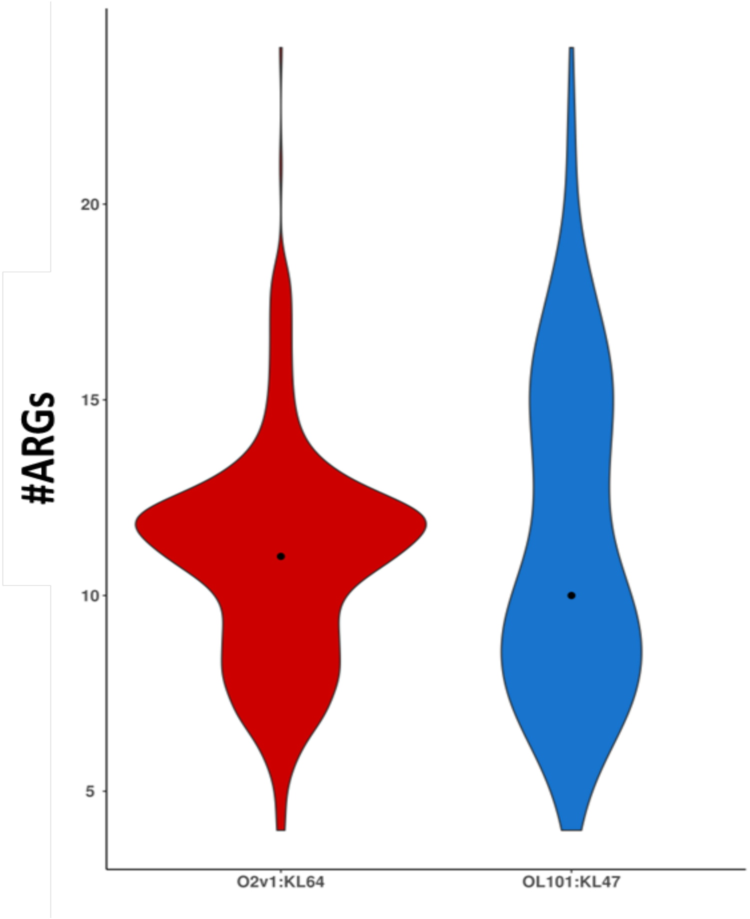

Fig. S10. Distribution of ARGs in O2v1:KL64 and OL101:KL47. No significant differences were detected for the number of ARGs between the two groups by Wilcoxon Rank Sum test (p = 0.99).

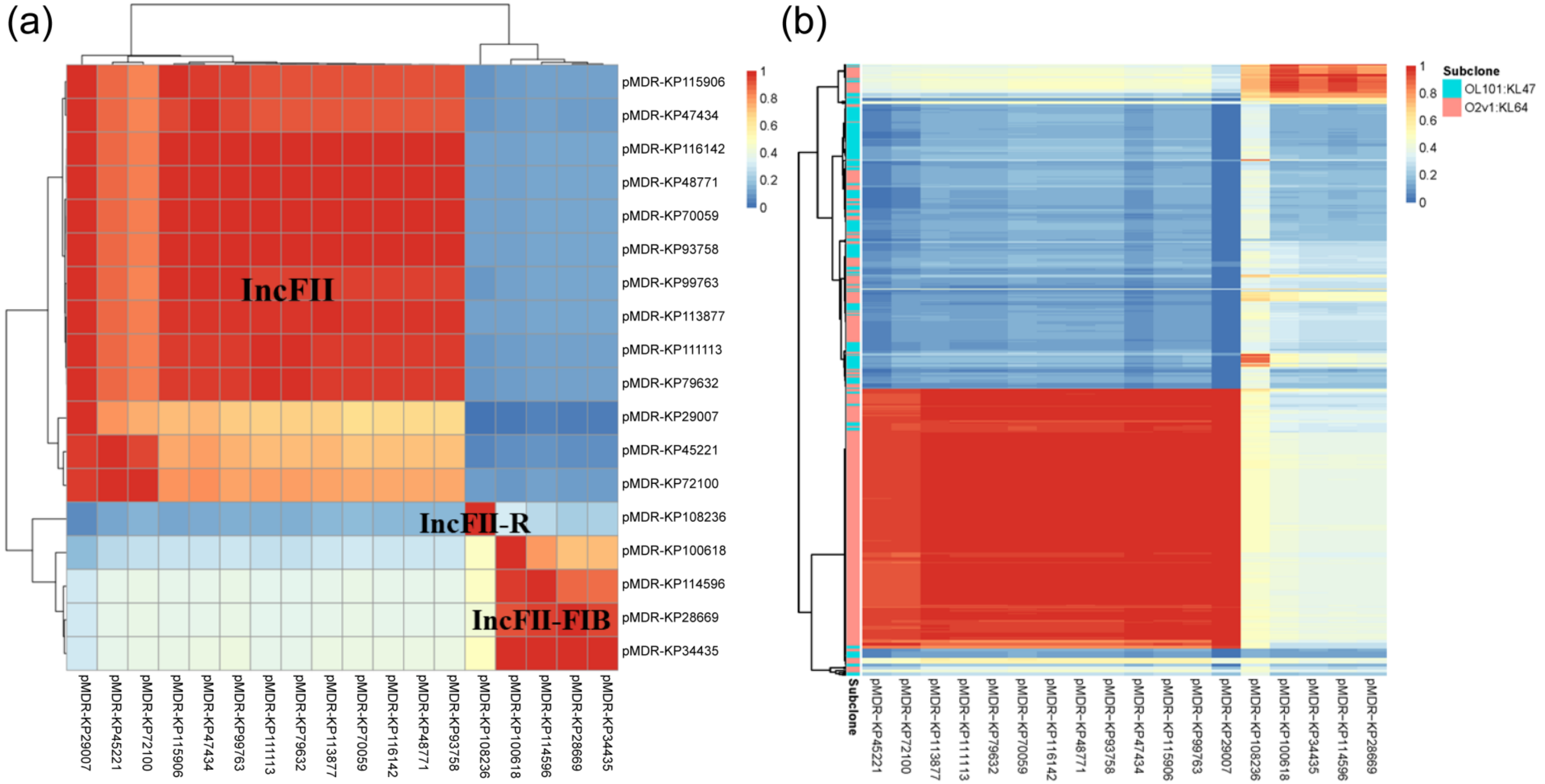

Fig. S11. Distribution of MDR plasmids carrying at least two of *qnrS1, bla*$_{\text{LAP-2}}$, *tet(A), dfrA*-like, *sul*-like genes in O2v1:KL64 and OL101:KL47. a, Comparison of the 18 putative MDR plasmids obtained by hybrid assemblies. The replicon type is correlated with the plasmid backbone similarity. **b,** The percentage length of putative MDR plasmid sequences was obtained by mapping short reads of the O2v1:KL64 and OL101:KL47 isolates to the 18 putative MDR plasmids.

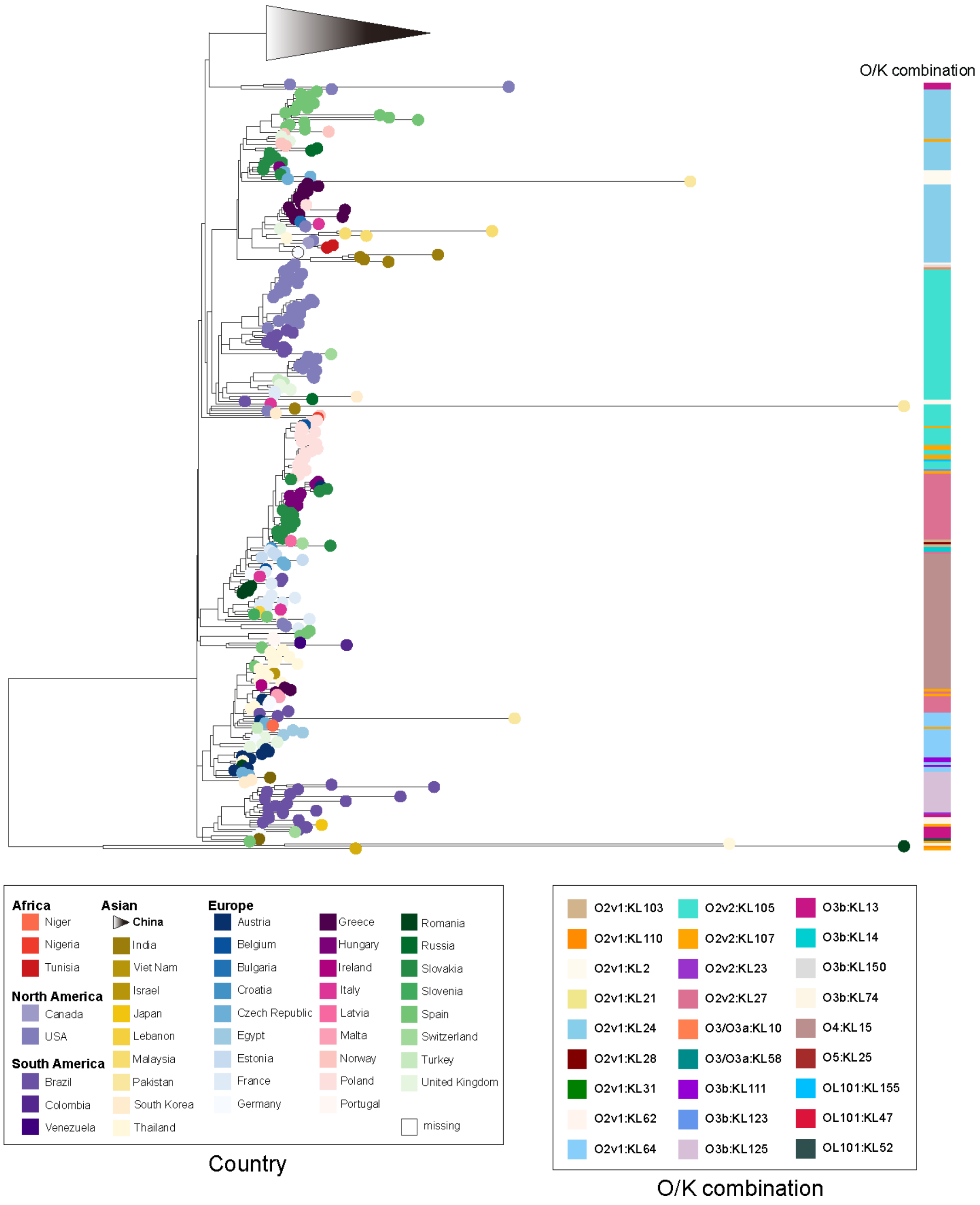

Fig. S12. International context of the ST11 clone. A phylogenetic tree of 975 ST11 genomes, comprising 646 genomes sequenced in this study, and 329 publicly available draft genomes collected in 43 countries across four continents (i.e. America, Africa, Asia and Europe) (Supplementary Table 9). The tree was mid-point rooted. Tips are coloured by the country of isolation and the metadata column shows the O- and K-type. The clade containing isolates from China is represented by the triangle.

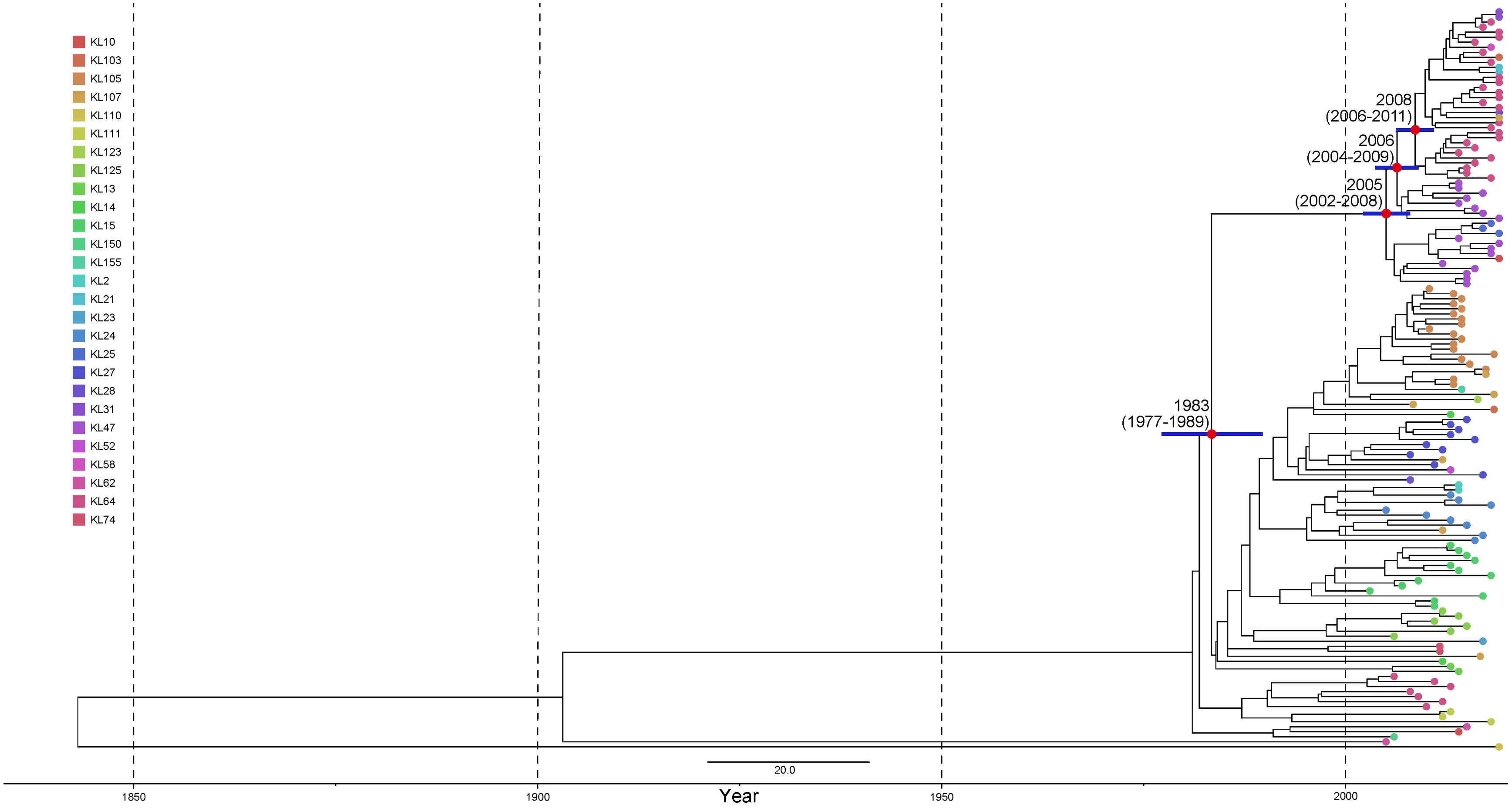

Fig. S13. BEAST chronogram of the ST11 clone. BEAST analysis was performed using 147 ST11 genomes, and the choice of the genomes is described in Methods. The figure shows a maximum clade credibility tree. MRCAs of key sublineages are indicated with 95% HPD bars.

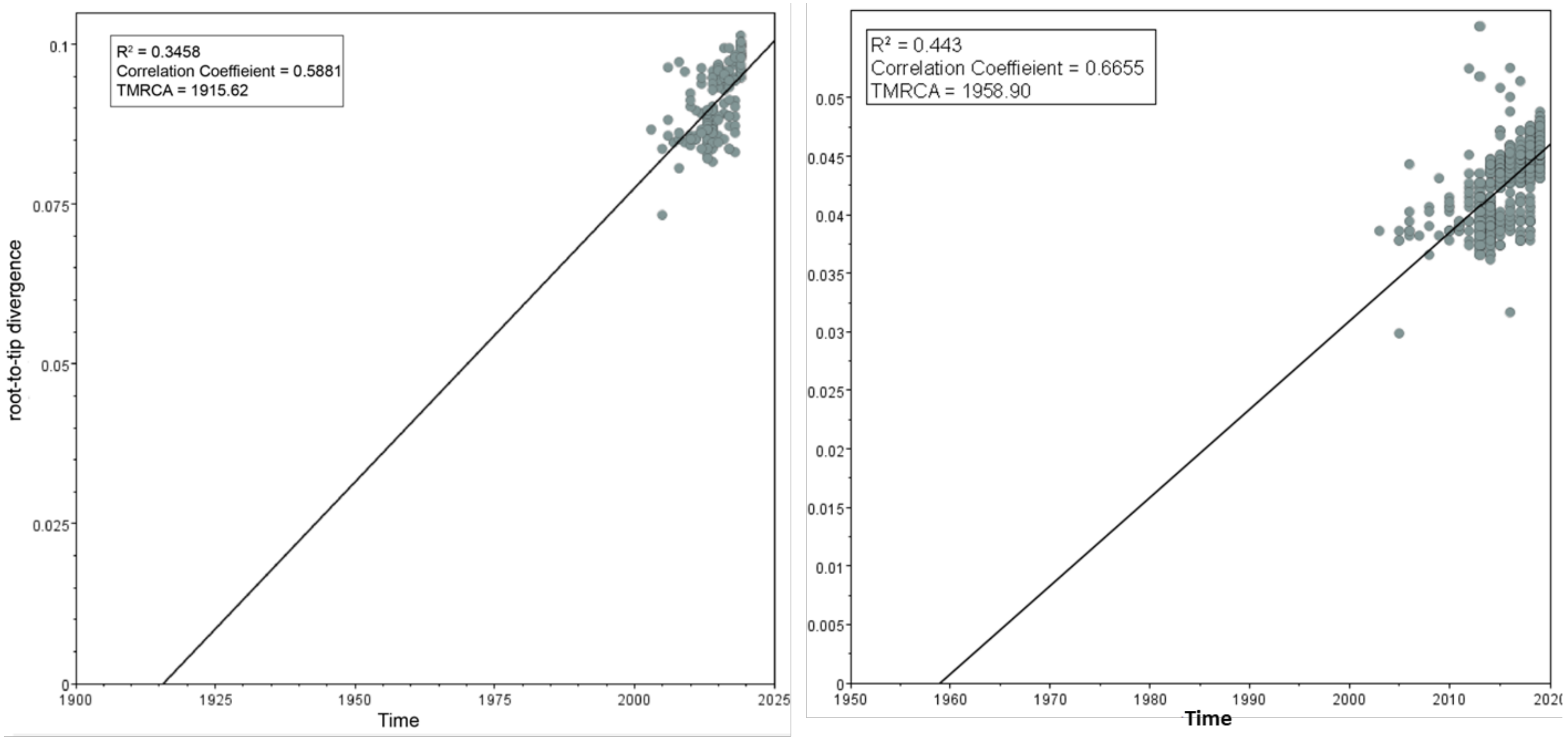

Fig. S14. Regression of the root-to-tip genetic distance against year of sampling for the ST11 genomes included in BEAST analysis. Two dataset were used for the analysis. The left panel includes 147 representative genomes, and the right panel includes all of the 975genomes. Both dataset support a relatively clocklike pattern of molecular evolution.

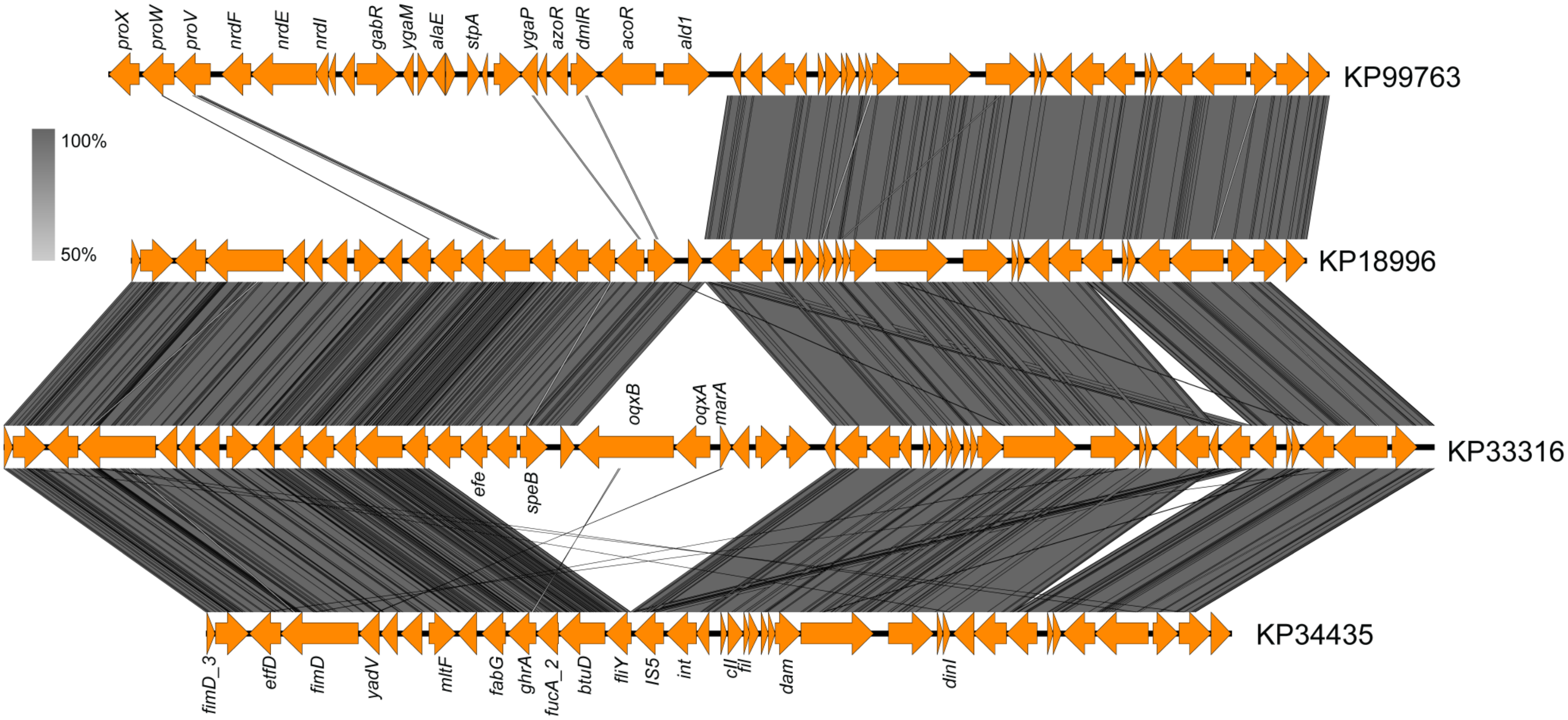

Fig. S15. Detection of the loss event of the *oqxAB* genes in O2v1:KL64 by synteny analysis. Four representative O2v1:KL64 isolates are shown. The fragments analyzed are derived from the long-read sequencing genomes.
