# Supplementary material for: A point mutation in recC associated with subclonal replacement of carbapenem-resistant Klebsiella pneumoniae ST11 in China
Source: Nat Commun. 2023 Apr 28;14:2464. doi: 10.1038/s41467-023-38061-z (PMC10147710; doi:10.1038/s41467-023-38061-z)
Supplement: Supplementary file 3 — Description to Additional Supplementary Information [file 41467_2023_38061_MOESM3_ESM.pdf]

Supplementary Dataset 1. Metadata and Kleborate analysis results of 794 CRKP collected in this study

Supplementary Dataset 2. Blasting the nucleotide sequence of recC-2804A>G against ST11-O2V1:KL64 genomes retrieved from GenBank

Supplementary Dataset 3. Abundance of 154 VFs in OL101:KL47 and O2v1:KL64

Supplementary Dataset 4. Mapping results of 646 ST11 genomes to pVir-KP47434 and pVir-KP16932

Supplementary Dataset 5. Hybrid assembly quality of 33 genomes showing coverage > 40% to pVir-KP47434 and/or pVir-KP16932.

Supplementary Dataset 6. Characteriazation of putative virulence plasmids deteted in 33 genomes

Supplemental Dataset 7. Abundance of ARGs in OL101:KL47 and O2v1:KL64

Supplementary Dataset 8. Hybrid assemly quality and characteriazation of 18 qnr-positive genomes

Supplementary Dataset 9. The charateriazation of ST11 genomes retrived from GenBank

Supplementary Dataset 10. Primers used in this study
