# Supplementary material for: A point mutation in recC associated with subclonal replacement of carbapenem-resistant Klebsiella pneumoniae ST11 in China
Source: Nat Commun. 2023 Apr 28;14:2464. doi: 10.1038/s41467-023-38061-z (PMC10147710; doi:10.1038/s41467-023-38061-z)
Supplement: Supplementary file 14 — Reporting Summary [file 41467_2023_38061_MOESM14_ESM.pdf]

nature portfolio

Corresponding author(s): Kai Zhou, Yonghong Xiao

Last updated by author(s): Apr 6, 2023

Reporting Summary

Nature Portfolio wishes to improve the reproducibility of the work that we publish. This form provides structure for consistency and transparency in reporting. For further information on Nature Portfolio policies, see our Editorial Policies and the Editorial Policy Checklist.

Statistics

For all statistical analyses, confirm that the following items are present in the figure legend, table legend, main text, or Methods section.

n/a	Confirmed	
☐	☒	The exact sample size (*n*) for each experimental group/condition, given as a discrete number and unit of measurement
☐	☒	A statement on whether measurements were taken from distinct samples or whether the same sample was measured repeatedly
☐	☒	The statistical test(s) used AND whether they are one- or two-sided<br>*Only common tests should be described solely by name; describe more complex techniques in the Methods section.*
☒	☐	A description of all covariates tested
☒	☐	A description of any assumptions or corrections, such as tests of normality and adjustment for multiple comparisons
☐	☒	A full description of the statistical parameters including central tendency (e.g. means) or other basic estimates (e.g. regression coefficient) AND variation (e.g. standard deviation) or associated estimates of uncertainty (e.g. confidence intervals)
☐	☒	For null hypothesis testing, the test statistic (e.g. *F*, *t*, *r*) with confidence intervals, effect sizes, degrees of freedom and *P* value noted<br>*Give P values as exact values whenever suitable.*
☐	☒	For Bayesian analysis, information on the choice of priors and Markov chain Monte Carlo settings
☒	☐	For hierarchical and complex designs, identification of the appropriate level for tests and full reporting of outcomes
☐	☒	Estimates of effect sizes (e.g. Cohen's *d*, Pearson's *r*), indicating how they were calculated

Our web collection on statistics for biologists contains articles on many of the points above.

Software and code

Policy information about availability of computer code

Data collection	No software was used
Data analysis	Trimmomatic v0.33; SPAdes v3.12.0; Unicycler 0.4.0; QUAST v4.6.0; FastANI v1.33; Prokka v1.14.6; Kleborate v2.0.1; Kaptive v1.0; Abricate v1.0.1; ResFinder v4.0; BacMet database v2.0; Virulence Factor Database 2019; BLASTp v2.6.0; IntegronFinder v1.5.1; TPMCalculator v0.0.3; phigaro v2.2.6; phytools v.0.4-98; Gubbins v2.2; BWA mem 62 v0.7.10-r789; SAMtools suite v1.7; GATK v3.7; Picard v2.10.1-SNAPSHOT; Bcftools v1.9-80; RAxML-ng v0.6.0; Snippy v4.6.0; BEAST v1.10.4; Tracer v1.7.2; FigTree v1.4.4; TempEst v1.5; TreeAnnotator v1.10; BLASTn v2.9.0; MOB-suite v3.0.3; Artemis Comparison Tool v13.0.0; BRIG v0.95; regentrans R package v1.0.0; pheatmap R package v1.0.12; Perl v5.26.2; Python v3.9.7; R v4.1.1Custom code used in this study is freely available at https://github.com/xuechunxu/CRKP_ST11_KL64

For manuscripts utilizing custom algorithms or software that are central to the research but not yet described in published literature, software must be made available to editors and reviewers. We strongly encourage code deposition in a community repository (e.g. GitHub). See the Nature Portfolio guidelines for submitting code & software for further information.

Data

Policy information about availability of data

All manuscripts must include a data availability statement. This statement should provide the following information, where applicable:

- Accession codes, unique identifiers, or web links for publicly available datasets
- A description of any restrictions on data availability
- For clinical datasets or third party data, please ensure that the statement adheres to our policy

All assembled Illumina sequence data have been deposited in GenBank under the BioProject accession number PRJNA778807 (https://www.ncbi.nlm.nih.gov/bioproject/PRJNA778807/). Individual accession numbers are also available in Supplementary Table 1. Source data are provided with this paper.

Human research participants

Policy information about studies involving human research participants and Sex and Gender in Research.

Reporting on sex and gender	NA
Population characteristics	NA
Recruitment	NA
Ethics oversight	NA

Note that full information on the approval of the study protocol must also be provided in the manuscript.

Field-specific reporting

Please select the one below that is the best fit for your research. If you are not sure, read the appropriate sections before making your selection.

☐ Life sciences ☐ Behavioural & social sciences ☒ Ecological, evolutionary & environmental sciences

For a reference copy of the document with all sections, see nature.com/documents/nr-reporting-summary-flat.pdf

Ecological, evolutionary & environmental sciences study design

All studies must disclose on these points even when the disclosure is negative.

Study description	Genomic analysis of 794 non-repetitive carbapenem-resistant Klebsiella pneumoniae (CRKP) bloodstream isolates submitted during the Survey of Blood Bacterial Resistant Investigation Collaborative System (BRICS).
Research sample	Between January 2014 and December 2019, 4,635 K. pneumoniae species complex bloodstream isolates were collected during the BRICS survey from 45 sentinel hospitals distributed across 19 provinces across China. A total of 794 non-repetitive CRKP bloodstream isolates were identified in 40 hospitals of 16 provinces.
Sampling strategy	All isolates collected during the BRICS survey were used, except where there was a lack of growth upon re-culture, contamination of the culture plates, or multiple phenotypes suggestive of contamination with other species.
Data collection	Isolates were collected by the staff of clinical laboratory of individual hospitals participating in the BRICS survey, and were sent to the central laboratory quarterly. The information of submitted isolates were recorded using Excel by the staff of clinical laboratory, and were sent to the coordinator of BRICS by Email.
Timing and spatial scale	Between January 2014 and December 2019, each participating hospital was asked to collect and provide the first blood isolate of each species per patient.
Data exclusions	Samples that were submitted during BRICS that were found to have a lack of growth, contamination in the culture plates, or multiple phenotypes, were not sequenced. These exclusion criteria were pre-established.
Reproducibility	Reproducibility is inherent to short-read sequencing provided that the sequencing coverage is high enough and the sequenced samples are not contaminated. To ensure reproducibility, we therefore applied rigorous QC filters to the sequence data as described in the Methods. All attempts at replication were successful.
Randomization	All submitted CRKP isolates were included in this study except for those failing QC criteria (i.e. random sampling did not apply).
Blinding	Blinding was not relevant because sources of bias that could be avoided by blinding did not play a role in our investigation, i.e. all results were objectively derived from genomic analysis and/or experiments.

Did the study involve field work? ☐ Yes ☒ No

Reporting for specific materials, systems and methods

We require information from authors about some types of materials, experimental systems and methods used in many studies. Here, indicate whether each material, system or method listed is relevant to your study. If you are not sure if a list item applies to your research, read the appropriate section before selecting a response.

Materials & experimental systems

n/a	Involved in the study
☒	☐ Antibodies
☒	☐ Eukaryotic cell lines
☒	☐ Palaeontology and archaeology
☒	☐ Animals and other organisms
☒	☐ Clinical data
☒	☐ Dual use research of concern

Methods

n/a	Involved in the study
☒	☐ ChIP-seq
☒	☐ Flow cytometry
☒	☐ MRI-based neuroimaging
